# Clinical Characteristics of Cryoglobulinemia With Cardiac Involvement in a Single Center

**DOI:** 10.3389/fcvm.2021.744648

**Published:** 2022-01-13

**Authors:** Kun He, Yun Zhang, Wei Wang, Yu Wang, Yue Sha, Xuejun Zeng

**Affiliations:** ^1^Department of Gastroenterology, Peking Union Medical College Hospital, Chinese Academy of Medical Sciences and Peking Union Medical College, Beijing, China; ^2^Department of Family Medicine & Division of General Internal Medicine, Department of Medicine, Peking Union Medical College Hospital, Chinese Academy of Medical Sciences, State Key Laboratory of Complex Severe and Rare Diseases (Peking Union Medical College Hospital), Beijing, China; ^3^Department of Hematology, Peking Union Medical College Hospital, Chinese Academy of Medical Sciences and Peking Union Medical College, Beijing, China

**Keywords:** cryoglobulinemia, cardiac involvement, clinical characteristics, treatment outcome, retrospective study

## Abstract

**Background:** Cryoglobulinemia is a syndrome characterized by the presence of cryoglobulins (CGs) in serum, and cardiac involvement is a rare occurrence that can affect treatment and prognosis. This study aimed to explore the clinical characteristics of cryoglobulinemia with cardiac involvement.

**Methods:** 108 patients diagnosed with cryoglobulinemia who were admitted and treated in Peking Union Medical College Hospital (PUMCH) between June 1985 and June 2019 were enrolled in the present study. Clinical characteristics, therapy, and prognosis of patients with cardiac involvement were retrospectively analyzed.

**Results:** The cryoglobulinemia with cardiac involvement was found in 7 patients, thus reaching the incidence of 6.5%. Heart failure was the main cardiac manifestation found in these patients, all with the involvement of external cardiac organs. Laboratory examinations showed significant elevation of N-terminal brain natriuretic peptide precursor (NT-proBNP) and brain natriuretic peptide (BNP) with negative troponin (cTnI). Electrocardiogram (ECG) was generally normal or only showed low-flat and biphasic multi-lead T waves. Echocardiography was performed in 6 patients, all of whom showed enlargement of heart cavity. Five patients had reduced left ventricular myocardial contractible motion with decreased ejection fraction, 3 patients had pericardial effusion, and 1 patient had left ventricular hypertrophy or severe aortic insufficiency. Cardiac magnetic resonance imaging showed delayed myocardial enhancement in 2 patients. One patient underwent a myocardial biopsy, which showed perivasculitis. Condition in 6 patients who received active treatment targeting improved in the early stage. Three patients (3/7, 42.9%) died due to disease progression during follow-up period.

**Conclusions:** Cryoglobulinemia with cardiac involvement is a rare but serious condition that has relatively high risk of death. When patients with cryoglobulinemia without underlying heart disease experience heart failure, chest pain, or elevation of asymptomatic NT-proBNP and BNP, there is a high possibility of cardiac involvement, even if the electrocardiogram and troponin are negative. Further examinations such as echocardiography, cardiac magnetic resonance imaging, and myocardial biopsy examination could contribute to the diagnosis. Cardiac manifestations could be timely reversed after active targeted treatment. NT-proBNP and echocardiography could be used for the monitoring of disease efficacy.

## Introduction

Cryoglobulinemia is a syndrome characterized by the presence of cryoglobulins (CGs) in the blood. Its pathological mechanism includes small vessel vasculitis caused by the deposition of immune complexes on the blood vessel wall and activation of complement. According to the composition of immunoglobulins, cryoglobulins can be divided into three types: Type I, a monoclonal immunoglobulin (usually IgG or IgM, rarely IgA or free light chain) that can be seen in malignant tumors with B cell lineage; Type II, which is a mixed type of monoclonal IgM and polyclonal IgG that can be seen in hepatitis C and other infections, connective tissue diseases (CTDs) and lymphoproliferative diseases; Type III, which is polyclonal IgM and polyclonal IgG, and usually appears secondary to CTDs or infection. Type II and III CGs are also known as mixed cryoglobulinemia. Cryoglobulinemia can cause multi-system diseases, including skin and mucosal injury, glomerulonephritis, peripheral nerve, joint pain, etc. Laboratory tests usually reveal positive monoclonal immunoglobulins, unexplained high titer rheumatoid factor (RF), and persistent hypocomplementemia. Therefore, the determination of blood cryoglobulinemia contributes to diagnosis. Cardiac involvement is a rare occurrence that can affect treatment and prognosis (1). To improve the understanding of cryoglobulinemia with heart involvement, the clinical data of 7 patients with cryoglobulinemia with heart involvement treated in our hospital were retrospectively analyzed and summarized. Relevant literature was also reviewed.

## Materials and Methods

### Patients

We reviewed medical records from all inpatients treated in Peking Union Medical College Hospital (PUMCH) between June 1985 and June 2019. A total of 108 patients diagnosed with cryoglobulinemia were found. The inclusion criteria were: (1) cryoglobulin positivity (cyocrit > 1.0%) and a clinical diagnosis of cryoglobulinemia based on comprehensive clinical, physical and chemical, imaging and pathological data (2, 3); (2) age > 18 years old (male or female), and admission to the ward with complete inpatients medical records; (3) cardiac involvement defined as abnormal structure or function of the heart caused by cryoglobulinemia based on clinical symptoms, physcial examinations, laboratory tests, radiology examinations, biopsy, and exclusion of heart failure caused by non-cardiac diseases like renal insufficiency and other secondary heart diseases such as hypertension, coronary heart disease (4). The exclusion criteria were: the missing inpatient medical records; availability of only outpatient clinic medical records.

### Methods

We recorded general data, the clinical manifestations, laboratory tests, imaging and pathology data, therapy, outcomes of patients with heart involvement by retrospective analysis. Clinical response was defined by analyzing the course of cardiac involvement (clinical, biologic, and radiologic improvement) and referring to the Bermingham vasculitis activity score (BVAS) of systemic vasculitis. Relapse was defined as reappearing of clinical signs of active vasculitis in any organ after remission (5, 6). Follow-up was conducted by outpatient or telephone contact. The last follow-up was in May 2020.

### Statistical Analysis

Normal distribution of data was established by the Kolmogorov-Smirnov test, and data were presented as mean ± standard deviation (Mean ± SD). Descriptive statistical analysis was carried out by SPSS 20.0 statistical package (SPSS, Chicago, IL, USA).

## Results

### General Data

There were 7 patients (7/108, 6.5%) with cryoglobulinemia with cardiac involvement who were treated in different departments, including the department of Nephrology (3/7), the department of Hematology (3/7), and the Department of General Medicine (1/7, who was later transferred to the Department of Hematology for treatment). There were 4 male and 3 female patients, with the mean age of cryoglobulinemia onset of 45.6 ± 13.1 years old and the mean age of cardiac involvement of 46.1 ± 12.8 years old. General data are shown in Table 1.

**Table 1:** General data and clinical features of 7 patients in cryoglobulinemia with cardiac involvement.

**Patients**	**Gender**	**Time at diagnosis (y)**	**Age at diagnosis (y)**	**The onset age of cardiac involvement (y)**	**Inpatient department**	**Previous heart disease**	**Involved organs other than the heart**						
							**Skin**	**Peripheral nerve**	**Kidney**		**Gastrointestinal tract**	**Lung**	**Others**
									**Clinical syndrome**	**Renal biopsy**			
1	M	2015	25	25	Nephrology	–	–	–	RI; NS	EPGN	–	+	–
2	M	2015	39	41	Nephrology	–	Purpura	–	RI; NS	–	–	–	–
3	M	2014	66	66	Nephrology	Hypertension	Purpura	–	RI; CNS	EPGN	–	–	Pancytopenia
4	F	2016	52	52	Hematology	–	Purpura	–	RI; CNS	–	–	–	Two bloodlines decreased; multiple lymph nodes enlargement; splenomegaly
5	F	2016	41	43	Hematology	–	Purpura	–	RI; NS	–	–	–	Two bloodlines decreased; multiple lymph nodes enlargement; splenomegaly
6	F	2019	54	54	Internal medicine of general medicine	–	Purpura	+	RI; CNS	–	+	–	–
7	M	2016	42	42	Hematology	–	Purpura	–	RI; Proteinuria	–	–	–	–
**Patients**	**Secondary causes**	**Laboratory examination**					**Renal biopsy**
		**Cryoglobulin**	**M-Ig**	**C3 (0.73–1.46 g/L)**	**C4 (0.1–0.4 g/L)**	**Rheumatoid factor (IU/mL)**	
1	HBV	II	IgMκ	0.636	0.013	254	EPGN
2	–	II	NM	0.373	0.059	138	ND
3	–	III	–	0.358	0.004	228	EPGN
4	SBL	I	IgMκ	0.232	0.003	463	ND
5	MZBL	II	IgMκ	0.711	0.043	11,405	ND
6	IBL	III	IgMκ	0.944	0.001	366	ND
7	–	I	IgG k	N	N	NM	ND

*y, year; M, male; F, female; “–”, negative; “+”, positive; RI, Renal insufficiency; NS, Nephrotic syndrome; CNS, Chronic nephritis syndrome; EPGN, Endocapillary proliferative glomerulonephritis. HBV, Hepatitis B Virus; “–”, negative; SBL, Small B-cell lymphoma; MZBL, marginal zone B-cell lymphoma; IBL, Indolent B cell lymphoma not otherwise specified; M-Ig, Monoclonal immunoglobulin; RF, Rheumatoid factor; EPGN, Endocapillary proliferative glomerulonephritis; N, Normal but without specific value; NM, Not mentioned; ND, Not done*.

### Types and Etiology of Cryoglobulin

Type I cryoglobulin was found in 2 patients, including one secondary to B-cell lymphoma and the other without a definite secondary factor. Type II cryoglobulin was found in 4 patients, including 1 secondary to chronic viral hepatitis B, 2 to B-cell lymphoma, and 1 without a definite secondary factor. One patient had type III cryoglobulin and no definite secondary factor.

### Heart Manifestation

Heart manifestations are shown in Table 2. Among the 7 patients, only 1 patient had a history of hypertension. The main clinical manifestations were chest tightness, suffocation, and edema; 2 patients had heart symptoms as the first manifestation, and the remaining 4 patients experienced heart-related symptoms in the process of disease progression or recurrence. The values of cTnI in 7 cases were all within the normal range. The values of NT-proBNP and BNP were significantly increased, especially in 2 cases with values higher than the upper limit of normal detection.

**Table 2:** Cardiac related manifestations, treatment, and prognosis in 7 patients with cryoglobulinemia.

**Patients**	**Before treatment**								**Treatment**	**1-2 months after treatment**				**Prognosis**
	**cTnI (0–0.056 ug/L)**	**NT-ProBNP (0–125 ng/L)**	**BNP (0–100 ng/l)**	**ECG**	**Echocardiography**		**MRI**	**Cardiac biopsy**		**Cardiac manifestations**	**NT-ProBNP (0-125ng/L)**	**Echocardiography**		
					**LVEF**	**Others**						**LVEF**	**Others**	
1	0.023	32,826	ND	Low and flat T- waves	37%	LV enlargement; Decreased LV systolic motion; Moderate PCE	ND	ND	Prednisone; Entecavir	Better	6354	49%	LV enlargement; Decreased LV systolic motion; Micro-PCE	Echocardiography was close to normal 2 years after treatment
2	0.05	>35,000	>5,000	N	37%	Enlargement of LV and RH; The decreased systolic motion of LV and RV; Severe aortic and tricuspid insufficiency; Mild PCE	Patchy delayed enhancement of ventricular septum	ND	Prednisone; Cyclophosphamide;	Worsen	ND	ND	ND	Death
3	0.01	7,710	302	Low and flat T- waves	59%	LV hypertrophy; Whole heart enlargement; Moderate PCE	ND	ND	Prednisone; Cyclophosphamide; Plasma exchange	Better	ND	59%	Whole heart enlargement; Mild-moderate PCE	Recurrence and death
4	0.003	2,049	190	Low and flat T- waves	48%	LA enlargement; Decreased LV systolic motion; Moderate PCE	ND	ND	Rituximab; Prednisone	Better	808	49%	LA enlargement; Decreased LV systolic motion; Mild PCE	Recurrence and death
5	0.01	7,424	369	N	ND	ND	Linear delayed enhancement of basal segment of the ventricular septum	ND	Rituximab; Cyclophosphamide Prednisone (RCP chemotherapy)	Better	100	ND	ND	No recurrence
6	0.045	>35,000	>5,000	Bipolar T- waves	48%	LA enlargement; Decreased LV systolic motion; Mild PCE	ND	ND	Rituximab; Cyclophosphamide; Dexamethasone (DRC chemotherapy)	Better	906	ND	ND	No recurrence
7	Normal	13,000	ND	N	37%	LV enlargement Decreased LV systolic motion	No delayed enhancement	Perivascular monocyte infiltration	Recurrence after glucocorticoid shock → Bortezomib; Cyclophosphamide; Dexamethasone (BCD chemotherapy)	Better	1010	56%	N	No recurrence

*ECG, Electrocardiogram; LVEF, Left ventricular ejection fraction; LV, Left ventricular; PCE, pericardial effusion; RV, right ventricles; RH, right heart; LA, Left atrial; MRI, magnetic resonance imaging; N, normal; NM, Not mentioned; ND, Not done*.

**Figure 1:**
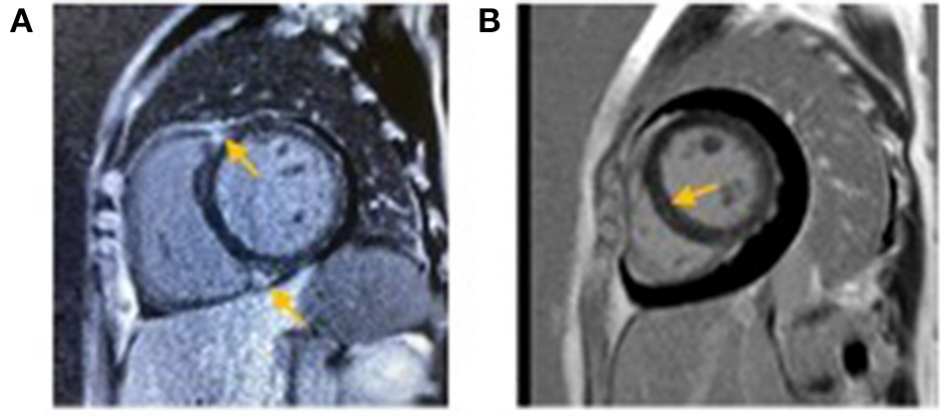
**(A)** Case 2 (Yellow arrow): Cardiac magnetic resonance imaging showing patchy delayed enhancement of the insertion at both ends of the ventricular septum. **(B)** Case 4 (Yellow arrow): Cardiac magnetic resonance imaging showing linear delayed enhancement in the basal segment of the ventricular septum.

Six cases underwent echocardiography, and the results were as follows: 6 cases had enlarged cardiac chambers, including left ventricular enlargement, with left and right ventricular enlargement and left atrial enlargement found in 2 cases; 5 cases had reduced left ventricular systolic motion and ejection fraction; 3 cases had pericardial effusion; 1 case had severe aortic insufficiency; 1 case had left ventricular hypertrophy with a history of hypertension. Three patients underwent cardiac magnetic resonance imaging. In addition to the corresponding findings of echocardiography, two patients had delayed myocardial enhancement in the ventricular septum (Figure 1). A myocardial biopsy was performed in 1 patient, revealing perivascular monocyte infiltration.

### Extracardiac Manifestations

In addition to cardiac involvement, renal involvement and renal insufficiency were present in all patients, including 3 cases of nephrotic syndrome, 3 cases of chronic nephritis syndrome, and 1 case with proteinuria in the nephrotic range. Renal biopsy was conducted in 1 case of nephrotic syndrome and the other case of chronic nephritis syndrome. Pathological results in both cases showed proliferative glomerulonephritis in capillaries, which could be consistent with renal involvement of cryoglobulinemia. The skin manifestations were purpura-like rash in all 6 cases. The lung, peripheral nerve, and gastrointestinal tract involvement were found in one case. There were 3 cases with decreased hemogram, including 1 case with unknown cause and the other 2 cases complicated with multiple lymph node enlargement and splenomegaly, which were considered secondary to lymphoma bone marrow involvement.

### Laboratory Tests

Increased rheumatoid factor and reduced complement were found in 6 cases. In 1 case, the complement was normal, and the result of a rheumatoid factor was unknown. Monoclonal immunoglobulin was detected in 6 cases, where 1 case was type III cryoglobulin negative, 4 cases had IgMk, and 1 case had IgGk.

### Treatment Plan and Efficacy

Two patients secondary to B-cell lymphoma received combined chemotherapy containing rituximab. One patient with type I cryoglobulin without definite secondary factors relapsed after receiving glucocorticoid pulse therapy in another hospital, and was later given chemotherapy containing bortezomib in our hospital. One patient with chronic hepatitis B was given high-dose glucocorticoid and antiviral therapy. The performance of heart and value of NT-proBNP in these 4 patients were significantly improved after receiving treatment. Early reexamination of echocardiography suggested that the heart structure and function were recovered; 1 patient showed normal echocardiography after 2 years of follow-up.

Four patients were followed up to the cut-off date without recurrence. One patient secondary to B-cell lymphoma (case 4) was treated with rituximab and high-dose glucocorticoid due to infection as a complication. As the above 4 patients, the cardiac indexes of this patient improved in the early stage. Nevertheless, the patient (case 4) died of recurrence after half a year later. The remaining 2 patients without definite secondary factors received immunosuppressive treatment with high-dose glucocorticoid and cyclophosphamide, where 1 patient received plasma exchange while the other did not. The former relapsed and died after experiencing a temporary improvement, while the latter died following the deterioration of the disease. Therefore, 3 patients (3/7, 42.9%) died due to disease progression during follow-up period.

## Discussion

Cryoglobulin is globulin that precipitates at the temperature below 37°C and re-dissolves at 37°C. This phenomenon was first observed in 1933, following which cryoglobulin was first defined in 1947. Afterward, it was gradually reported in many diseases (7, 8). Cryoglobulinemia, which is an uncommon condition, rarely includes cardiac involvement; however, it has been associated with a higher risk of death. Up to now, the cardiac involvement of cryoglobulinemia was mostly reported in individual cases, few of whom are from China. Herein, we retrospectively analyzed the data of cryoglobulinemia with heart involvement treated in our center to further understand this disease.

In a retrospective study of 165 patients with mixed cryoglobulinemia associated with hepatitis C conducted in 2013, Terrier et al. (4) found that 7 of patients had heart involvement (4.2%), with a median onset age of 61 years old (40–76), while the onset age of heart involvement was not mentioned. Currently, there is a lack of related research reports from China. This study found that the prevalence of cardiac involvement with cryoglobulinemia was approximately 6.5%, the mean onset age of cryoglobulinemia was 45.6 ± 13.1 years old, and the cardiac involvement was 46.1 ± 12.8 years old.

According to previous literature reports, almost all heart involvement in cryoglobulinemia reported so far were mixed type CGs. There was 1 case with heart involvement in type I cryoglobulinemia found in the hematology department of our center (9). Retrospective analysis of cases in our center revealed another case of type I cryoglobulinemia with cardiac involvement besides the previously mentioned one. The cryoglobulin of the remaining 5 cases with cardiac involvement was mixed type, including 4 cases of type II and 1 case of type III.

Among the 7 cases in this study, 6 had no underlying heart disease, and 1 had pre-existing hypertension. All patients presented with congestive heart failure, including acute pulmonary edema and edema around the body. Laboratory examinations indicated significantly increased NT-proBNP and BNP. The echocardiographic findings included decreased left ventricular myocardial systolic movement and left ventricular ejection fraction, cardiac cavity enlargement to varying degrees, left ventricular hypertrophy, pericardial effusion, and severe aortic valve insufficiency. Cardiac magnetic resonance imaging was performed in 3 cases, while 2 showed delayed myocardial enhancement, thus suggesting cardiomyopathy. No delayed myocardial enhancement was found on cardiac magnetic resonance imaging in the last case, which may be related to the previous application of high-dose glucocorticoids in other hospitals. One patient underwent a myocardial biopsy, which revealed perivasculitis. The clinical manifestations, laboratory indexes, and echocardiography of the heart all improved after treatment of cryoglobulinemia and secondary causes, which further supported the premise that the damage caused by heart diseases contributed to cryoglobulinemia. As previously mentioned in the study by Terrier et al. (4), there were 7 cases with heart failure and 4 cases with chest pain. Electrocardiogram showed T wave changes in 6 cases, ST-segment changes in 1 case, roughly normal condition in 1 case, while elevated troponin was found in 5 cases. Echocardiography revealed left ventricular hypertrophy in 1 case, pericardial effusion in 2 cases, myocardial contraction movement in 5 cases, and cardiac lumen enlargement in 6 cases. Myocardial MRI was performed in 5 patients, including 3 cases with delayed myocardial enhancement. In the present study, we found no chest pain, ST-segment abnormality, or elevated troponin. Although coronary artery was not evaluated in our study due to renal insufficiency, high risks of coronary atherosclerosis were not found in these cases which suggested cardiac damage was not considered to be caused by coronary lesions in a traditional way. Electrocardiogram results (close to normality or only multi-lead T wave change), negative troponin, and echocardiography without segmental ventricular wall motion abnormality further supported this assumption, which was also consistent with previous studies. For example, in Terrier's study (4), patients with ST-segment changes and troponin elevation underwent coronary angiography (CAG), which showed no obvious abnormality. Moreover, we found negative results for coronary evaluation of 3 patients in other foreign case reports (Table 3). Besides, Maestroni *et al*. conducted a study in (16), reporting on coronary artery vasculitis found after autopsy in two deceased patients with cryoglobulinemia with heart involvement. Therefore, we speculated that cryoglobulin-associated vasculitis might cause heart disease by mediating coronary microcirculation disturbance. In the present study, there was 1 case with left ventricular hypertrophy on echocardiography, which is consistent with previous studies mentioned above. Although the case in our study with left ventricular hypertrophy had pre-existing hypertension, it was still necessary to consider the possibility of left ventricular hypertrophy being secondary to cryoglobulinemia as it improved and disappeared after the treatment of the primary disease. This mechanism might be associated with inflammatory edema in myocardial involvement caused by cryoglobulinemia, which needed to be confirmed by myocardial biopsy. As for the severe valve disease mentioned in this study, the correlation between valve disease and cryoglobulinemia remains unclear as there were no follow-up echocardiography data (the patient died following the deterioration of the disease), and no related reports were recorded in previous studies. Yet, we assume that heart valve lesions observed in the present study might be related to cryoglobulinemia as the patient had no pre-existing heart disease and previous studies reported heart valve lesions in ANCA-related vasculitis, which equally belong to the category of small vessel vasculitis. A heart biopsy was also needed for definite confirmation (17).

**Table 3:** Cardiac involvement related to cryoglobulinemia reported in the literature.

**References**	**Age (y)**	**Gender**	**Clinical manifestations**	**Cardiac MRI**	**Coronary artery CTA**	**Myocardial biopsy**	**Cryoglobulin**	**Secondary causes**	**Treatment**	**After treatment**
Tulio et al. (10)	79	F	Dyspnea, Edema	ND	ND	ND	II	HCV and intestinal TB	Recurrence after glucocorticoid shock → Rituximab; Antiviral therapy	Better
Ali et al. (11)	45	M	Dyspnea, Edema	Pericarditis and myocarditis	ND	ND	(Cryoglobulinemia was confirmed by clinical and renal puncture)	HCV	Rituximab; Plasma exchange; Antiviral therapy	Better
Cavalli et al. (12)	65	M	Dyspnea, Edema	Decreased LV systolic motion; LV hypertrophy; Delayed enhancement of inferior and lateral walls of LV	–	ND	ND	HCV	Rituximab; Antiviral therapy	Better
Karras et al. (13)	63	F	Dyspnea, Edema	Decreased LV systolic motion and enlarged LV	–	ND	II	HCV	Rituximab; Antiviral therapy	Better
Ghijsels et al. (14)	44	M	Dyspnea, Edema	ND	–	ND	II	–	Glucocorticoid; Cyclophosphamide; Plasma exchange; Rituximab	Better
Culclasure et al. (15)	65	M	Dyspnea, Edema	ND	ND	ND	II	–	Glucocorticoid; Immunosuppressant (Unknown); Plasma exchange	Better

*y, years; F, female; M, male; MRI, magnetic resonance imaging; LV, Left ventricular; ND, Not done; “–”, negative; HCV, Hepatitis C Virus; TB, tuberculosis; Electrocardiogram and echocardiography data were not mentioned in the above literature*.

In the present study, skin and renal lesions were the most common extracardiac manifestations in patients with cardiac involvement. Laboratory examinations showed increased RF and decreased complement, while 1 case with normal complement may be due to the previous high-dose glucocorticoid treatment. These results were roughly consistent with previous studies (18, 19). In addition, Terrior et al. (4) compared patients with cryoglobulinemia to those without cardiac involvement and found 6-month, 1-, and 2-year survival rates of 86% vs. 99%, 71% vs. 96%, and 48% vs. 90%, respectively (HR 5.01, *P* = 0.003). This suggested that patients of cryoglobulinemia with cardiac involvement had a higher risk of death. This study also showed relatively high risk of death in patients with cardiac involvement as 3 patients (3/7, 42.9%) died due to disease progression during follow-up period. Therefore, clinicians need to pay greater attention to patients with cryoglobulinemia with cardiac manifestations. In a retrospective study of 54 patients with HCV-associated mixed cryoglobulinemia conducted in 2010, Antonelli et al. (20) found that elevated NT-proBNP in laboratory examination for patients with no cardiac manifestations may indicate the presence of subclinical cardiac damage. This seems to suggest that NT-proBNP may be a potential indicator for early identification of cardiac involvement, being cost-effective and convenient to detect. In addition, our results revealed that NT-proBNP and echocardiogram were also improved with the improvement of the cardiac clinical manifestations after treatment, thus suggesting that both of them could be used for monitoring the efficacy of diseases. By the way, BNP may have the same effect for monitoring as Nt-proBNP because they have the same origin, which needs dynamic data of it before and after treatment to confirm in the further study. And BNP may make more sense than NT-proBNP in patients with cardiac involvement and kidney disease at the same time as it was less affected by renal insufficiency.

Given the rarity of this disease and the lack of mass evidence-based medical evidence for the cardiac involvement of cryoglobulinemia, currently, there is no recognized treatment plan. Patients are mainly treated by hematologists or immunologists according to their personal experience, and the treatment philosophy may vary for each individual case. In addition to treating secondary causes of cryoglobulinemia, immunosuppressive therapy should be selected according to the scope and severity of target organ involvement. The main therapeutic modalities include high-dose glucocorticoids, cyclophosphamide, rituximab, and plasma exchange (21, 22), which is also roughly consistent with the treatment regimens received by the patients in the present study. Since the heart is an important target organ, active treatment should be given once the involvement of the heart is confirmed. In this study, patients were mainly treated in the Department of Nephrology and Department of Hematology. The ones treated at the former department mainly received glucocorticoids and immunosuppressive agents, while some patients needed combined plasmapheresis. The treatment at the latter department mainly focused on combination therapy with rituximab, which may be related to past treatment experience and secondary disease due to lymphoma. In terms of efficacy, available case reports and case series analyses, including the study conducted in our center, all suggested that the clinical symptoms, biochemical indexes, and imaging changes of cardiac lesions of the patients receiving regular and standardized treatment all significantly improved after early treatment, while the long-term prognosis still requires longer follow up. Notably, the condition of a heart-involvement patient with type I cryoglobulinemia without clear secondary etiology who was treated in our center by bortezomib-containing chemotherapy after relapse of high-dose glucocorticoid therapy significantly improved. To the best of our knowledge, this was the first case receiving bortezomib treatment for cryoglobulinemic cardiac involvement. From the perspective of mechanism, rituximab mainly targets B cells, while bortezomib mainly targets plasma cells. Both of them seem to have good efficacy in treating heart invvement with cryoglobulinemia, which may provide new ideas and references for the treatment of such patients.

The present study has the following limitations: firstly, this was a retrospective study; thus, there may be information bias. Secondly, as a single-center study, the number of included cases was small, and the follow-up time was short, so it was difficult to fully reflect the characteristics and prognosis of this disease. However, it is also difficult to obtain large-scale clinical data and conduct prospective studies due to the rarity of heart involvement with cryoglobulinemia.

## Conclusions

In this study, we retrospectively analyzed the clinical data of patients with cryoglobulinemia with heart involvement treated in our center and reviewed relevant literature, thus aiming to improve the understanding of clinicians on cryoglobulinemia with heart involvement as well as advance the early diagnosis and treatment of this kind of disease. Heart involvement in cryoglobulinemia is a rare occurrence that carries a relatively high risk of death. For patients with cryoglobulinemia, cardiac involvement should be considered when there are cardiac-related clinical manifestations or asymptomatic elevation of NT-proBNP and BNP. Electrocardiogram changes may be insignificant, and troponin can be negative in these patients. Echocardiography, cardiac magnetic resonance imaging, and myocardial biopsy can help detect the disease, and the heart condition can be reversed with early and targeted treatment. In addition, subsequent multi-center, prospective and large-sample-size study are needed to confirm the conclusions in this study, further clarify the viscera lesions, pathogenesis, and risk factors of cryoglobulinemia with cardiac involvement and conduct early detection, diagnosis, and early treatment.

## Data Availability Statement

The original contributions presented in the study are included in the article/supplementary material, further inquiries can be directed to the corresponding author.

## Ethics Statement

The studies involving human participants were reviewed and approved by the Committee of Peking Union Medical College Hospital. The patients/participants provided their written informed consent to participate in this study. Written informed consent was obtained from the individual(s) for the publication of any potentially identifiable images or data included in this article.

## Author Contributions

YZ: study conception and design. KH: literature review and data extraction. WW and XZ: quality control. KH: statistical analysis. KH, YZ, WW, YW, YS, and XZ: manuscript preparation. YZ: manuscript review. All authors contributed to the article and approved the submitted version.

## Funding

This study was supported by the National Natural Science Foundation of China (No. 81901667) and the 2019 Discipline Development Project of Peking Union Medical College (No.201920200106).

## Conflict of Interest

The authors declare that the research was conducted in the absence of any commercial or financial relationships that could be construed as a potential conflict of interest.

## Publisher's Note

All claims expressed in this article are solely those of the authors and do not necessarily represent those of their affiliated organizations, or those of the publisher, the editors and the reviewers. Any product that may be evaluated in this article, or claim that may be made by its manufacturer, is not guaranteed or endorsed by the publisher.
