# Corrigendum: Clinical Characteristics of Cryoglobulinemia With Cardiac Involvement in a Single Center

**DOI:** 10.3389/fcvm.2022.899282

**Published:** 2022-04-01

**Authors:** Kun He, Yun Zhang, Wei Wang, Yu Wang, Yue Sha, Xuejun Zeng

**Affiliations:** ^1^Department of Gastroenterology, Peking Union Medical College Hospital, Chinese Academy of Medical Sciences and Peking Union Medical College, Beijing, China; ^2^Department of Family Medicine & Division of General Internal Medicine, Department of Medicine, Peking Union Medical College Hospital, Chinese Academy of Medical Sciences, State Key Laboratory of Complex Severe and Rare Diseases (Peking Union Medical College Hospital), Beijing, China; ^3^Department of Hematology, Peking Union Medical College Hospital, Chinese Academy of Medical Sciences and Peking Union Medical College, Beijing, China

**Keywords:** cryoglobulinemia, cardiac involvement, clinical characteristics, treatment outcome, retrospective study

In the published article, affiliation 2 was incorrectly written as: “2 State Key Laboratory of Complex Severe and Rare Diseases (Peking Union Medical College Hospital), Department of Family Medicine & Division of General Internal Medicine, Department of Medicine, Peking Union Medical College Hospital, Chinese Academy of Medical Sciences, Beijing, China.” The correct affiliation is: “2 Department of Family Medicine & Division of General Internal Medicine, Department of Medicine, Peking Union Medical College Hospital, Chinese Academy of Medical Sciences, State Key Laboratory of Complex Severe and Rare Diseases (Peking Union Medical College Hospital), Beijing, China.”

The authors apologize for this error and state that this does not change the scientific conclusions of the article in any way. The original article has been updated.

## Publisher's Note

All claims expressed in this article are solely those of the authors and do not necessarily represent those of their affiliated organizations, or those of the publisher, the editors and the reviewers. Any product that may be evaluated in this article, or claim that may be made by its manufacturer, is not guaranteed or endorsed by the publisher.

